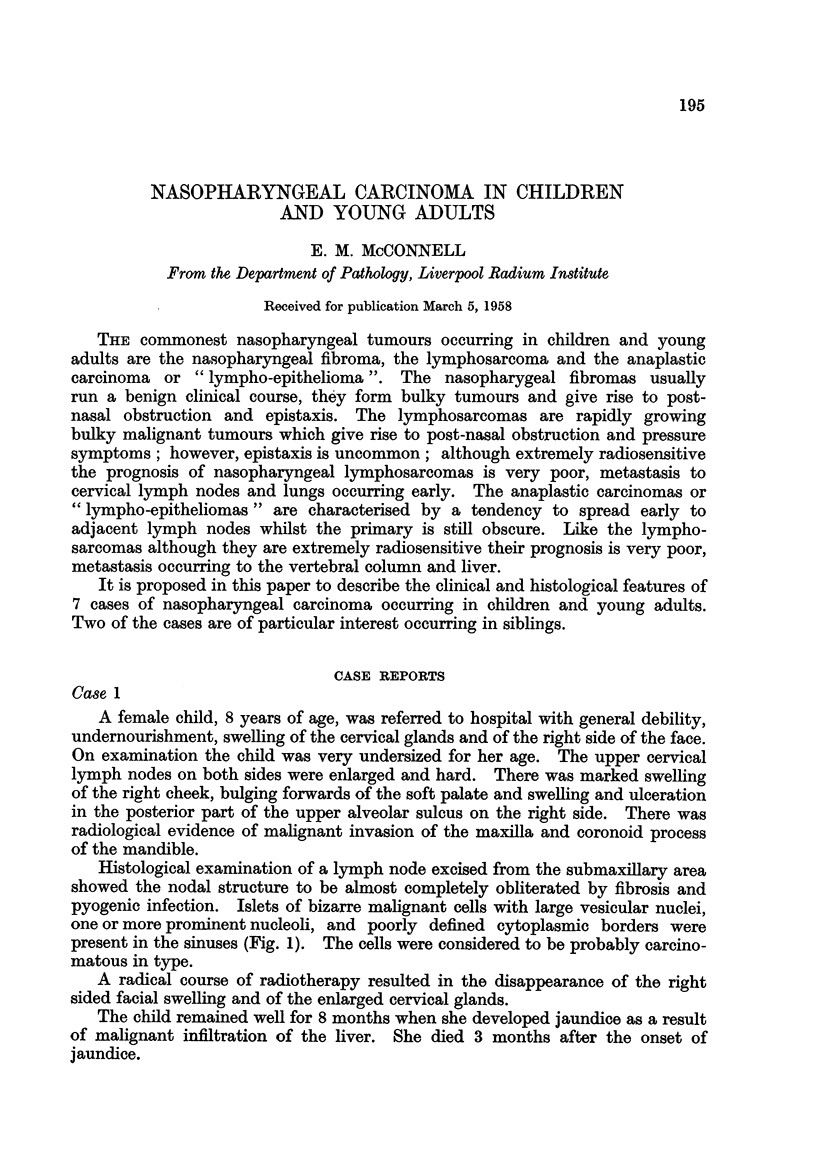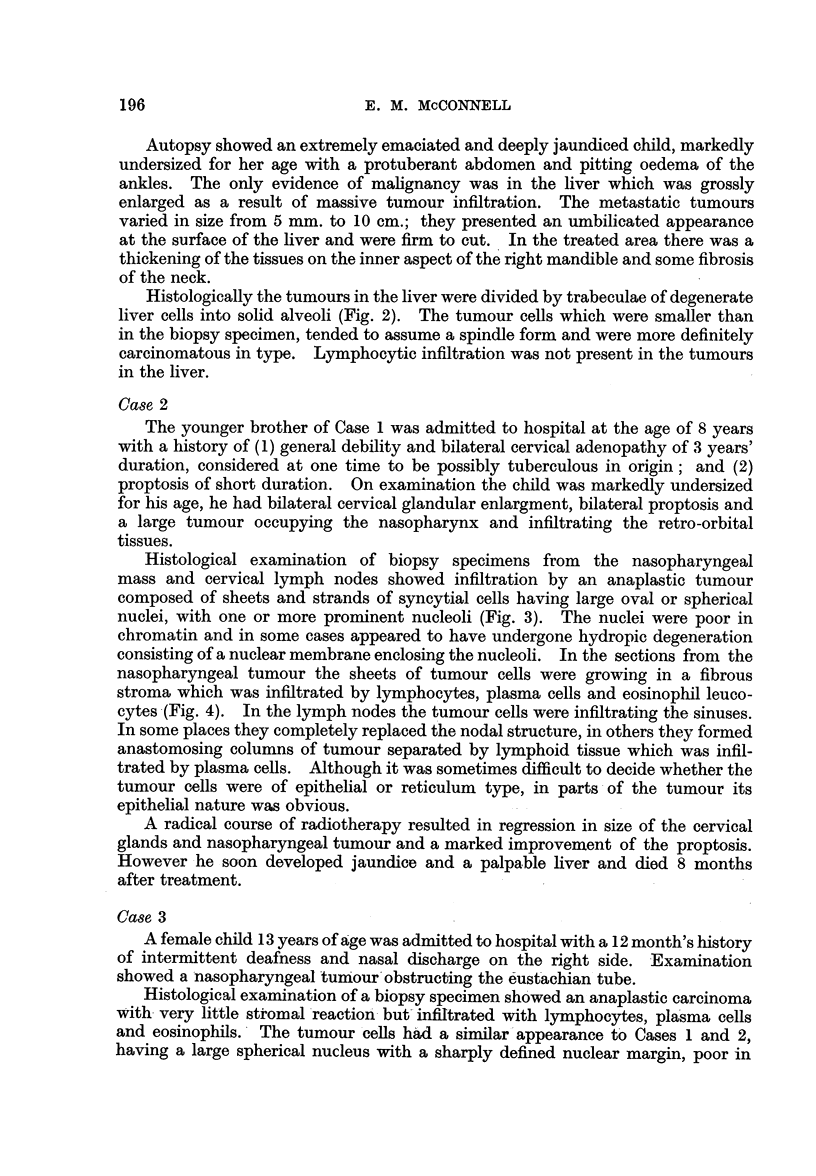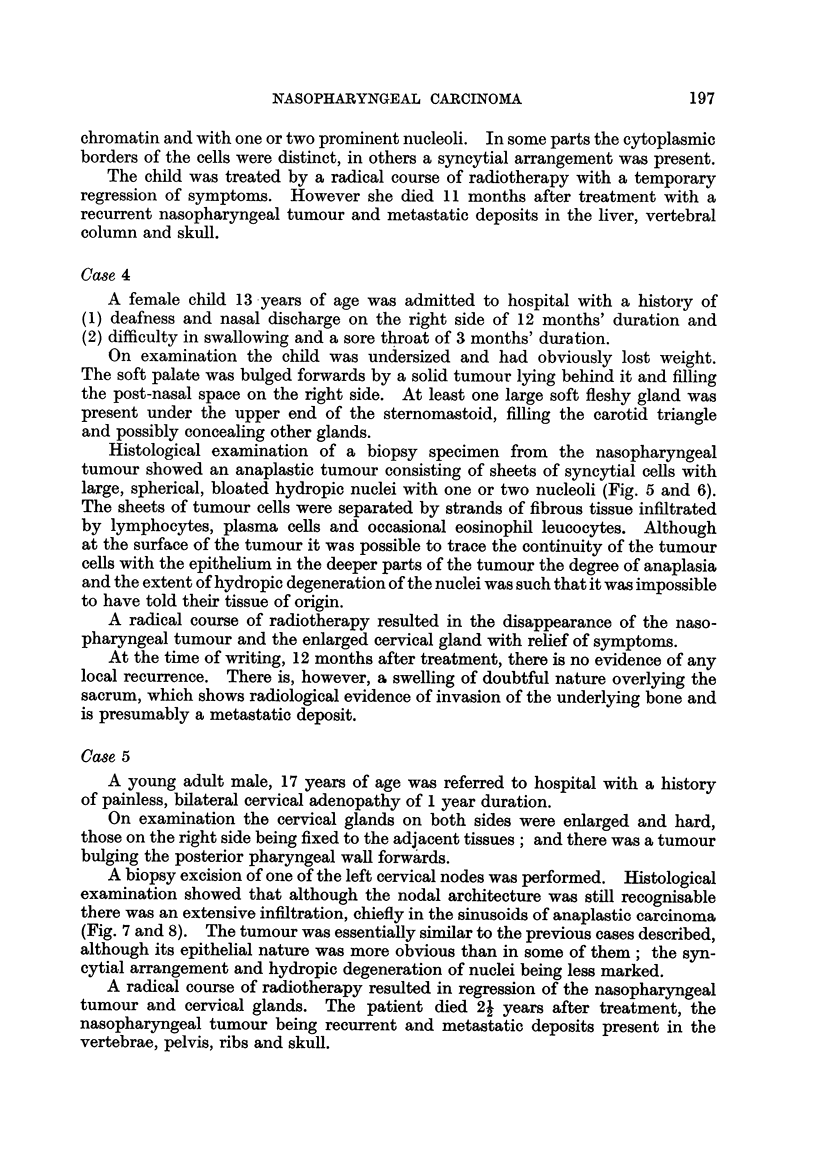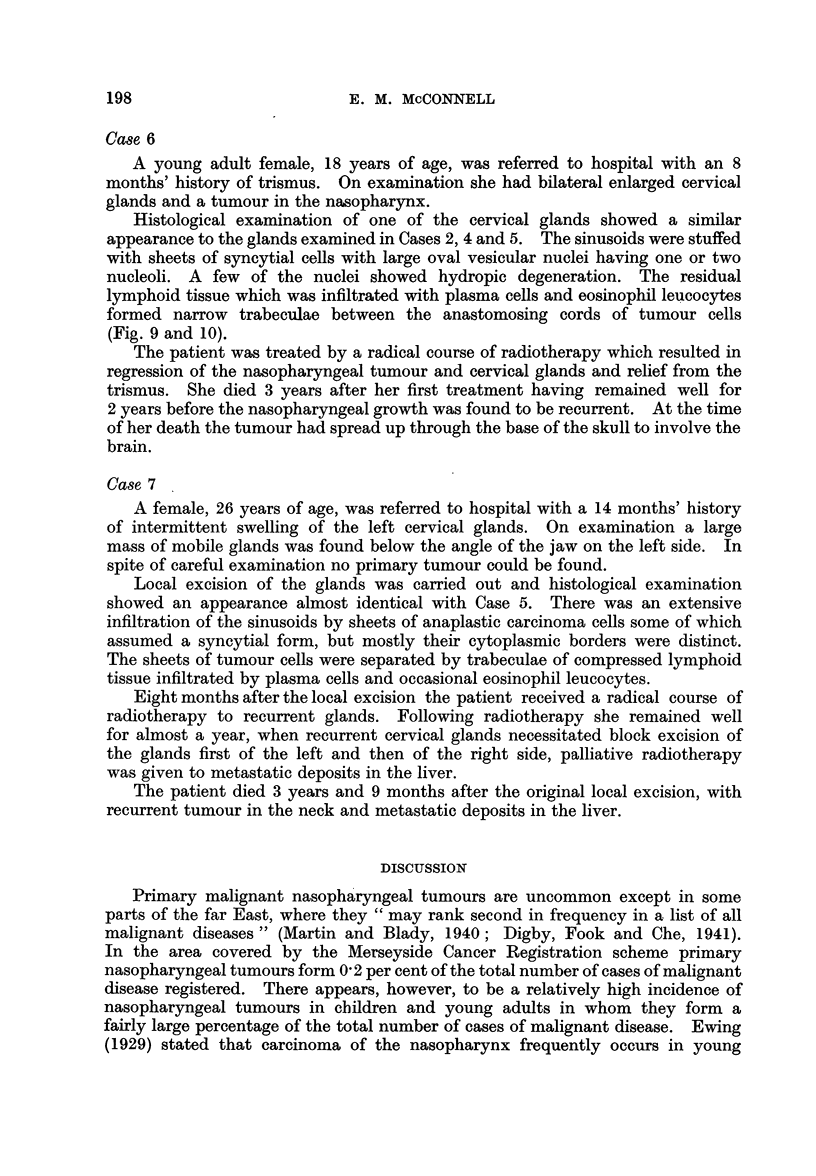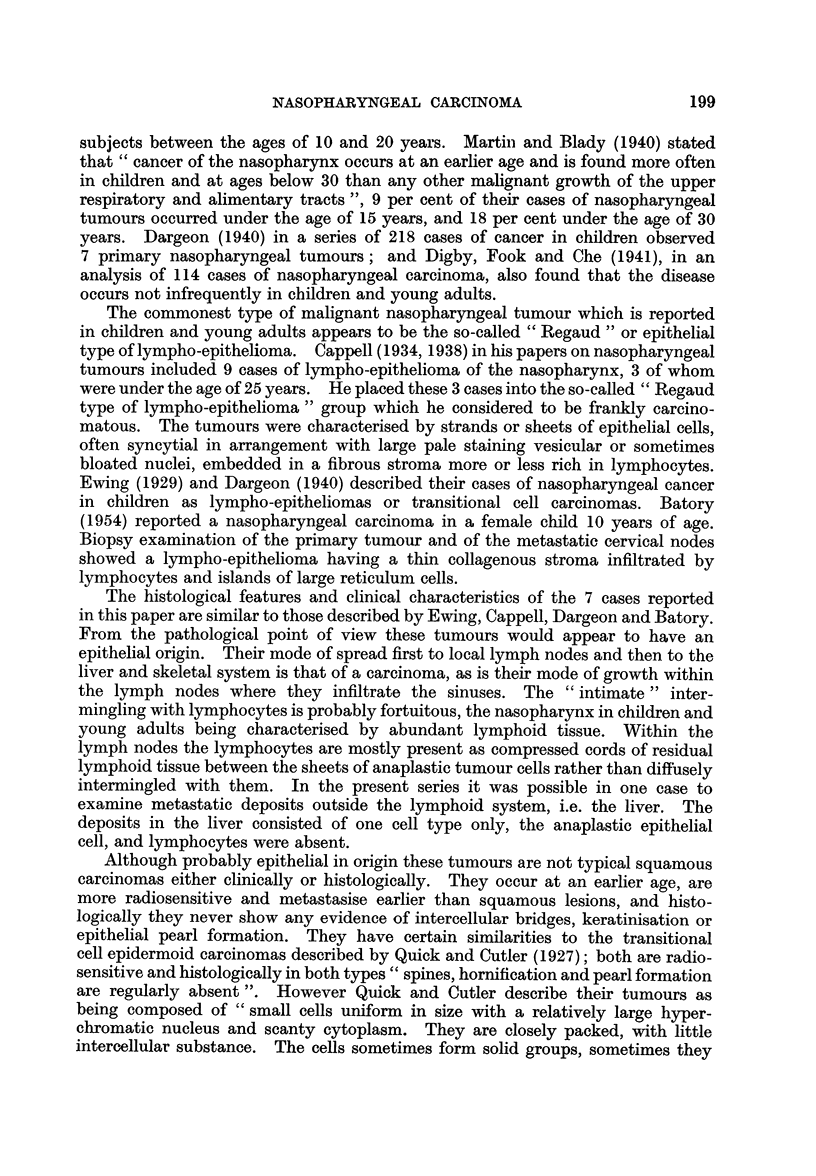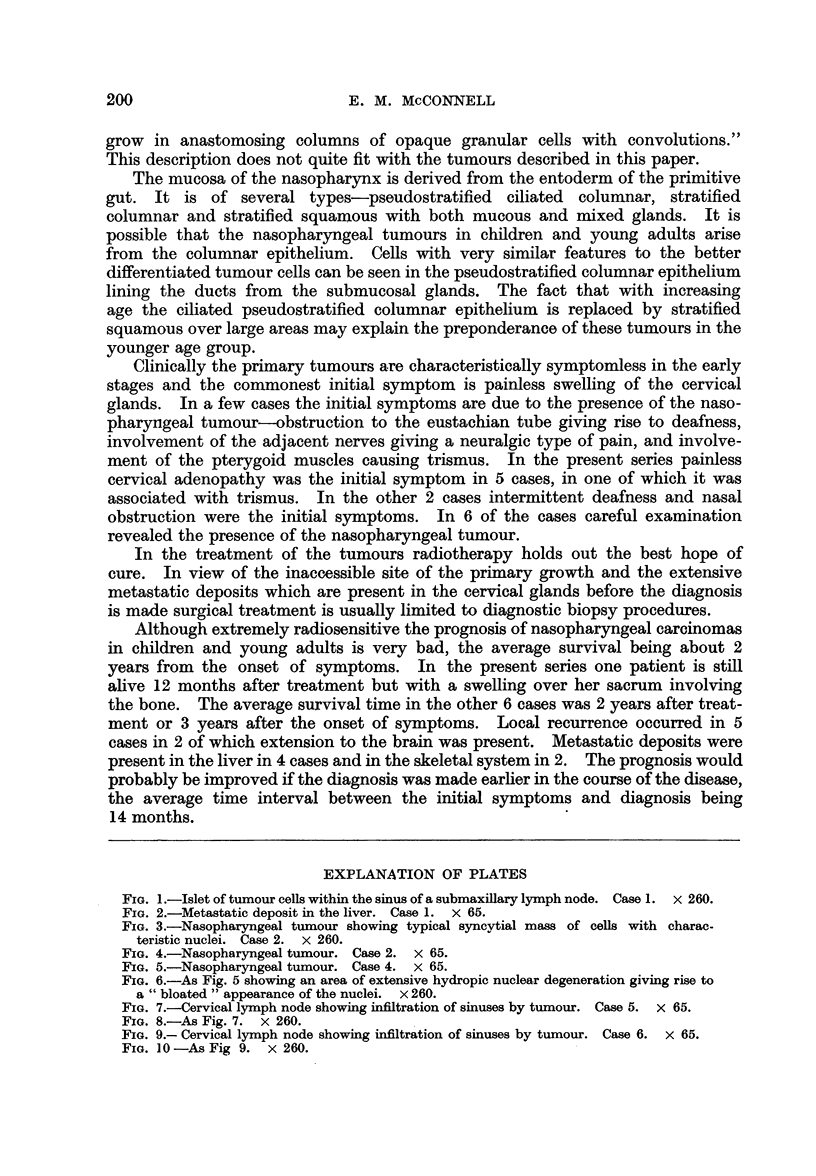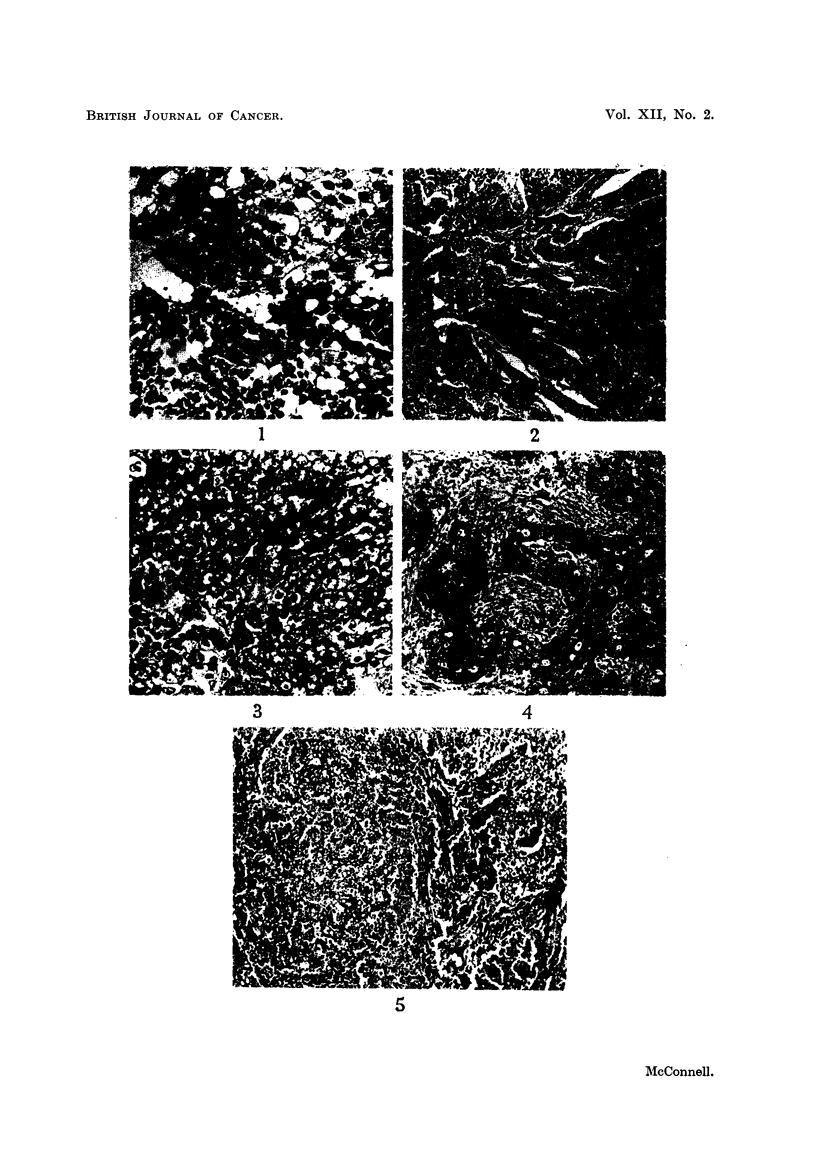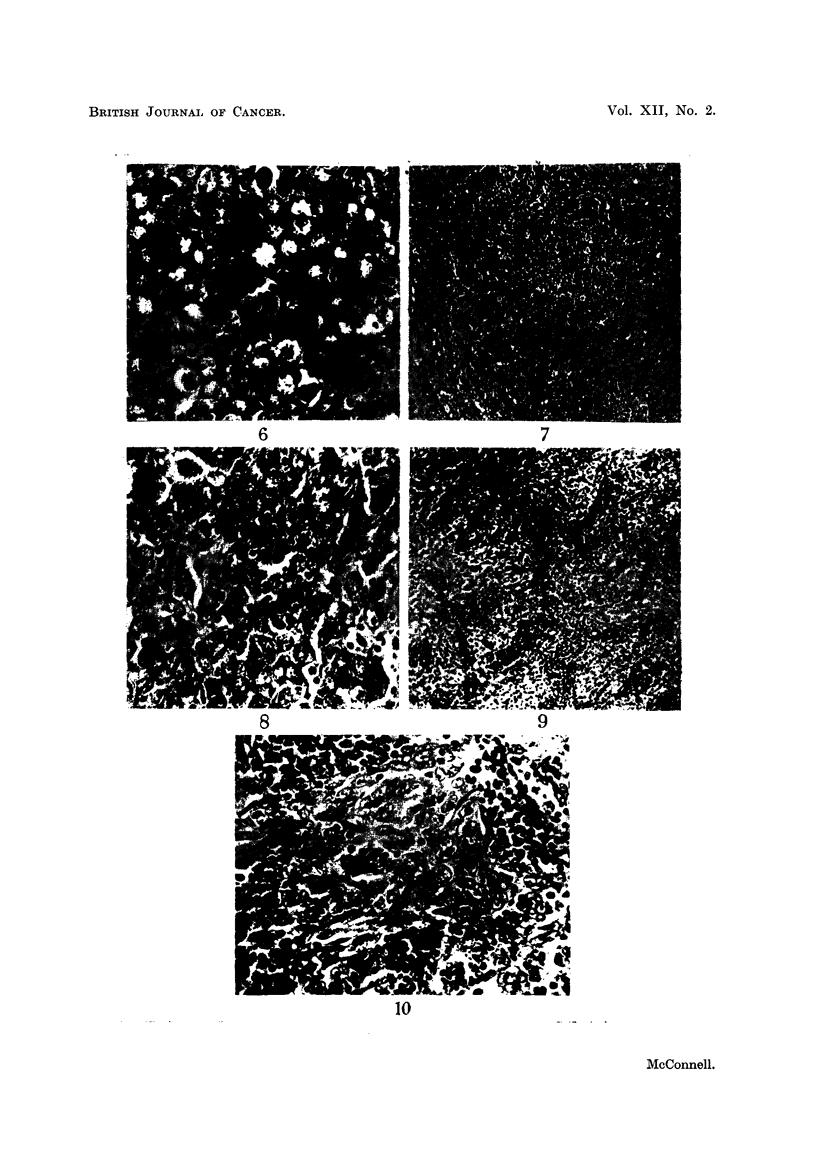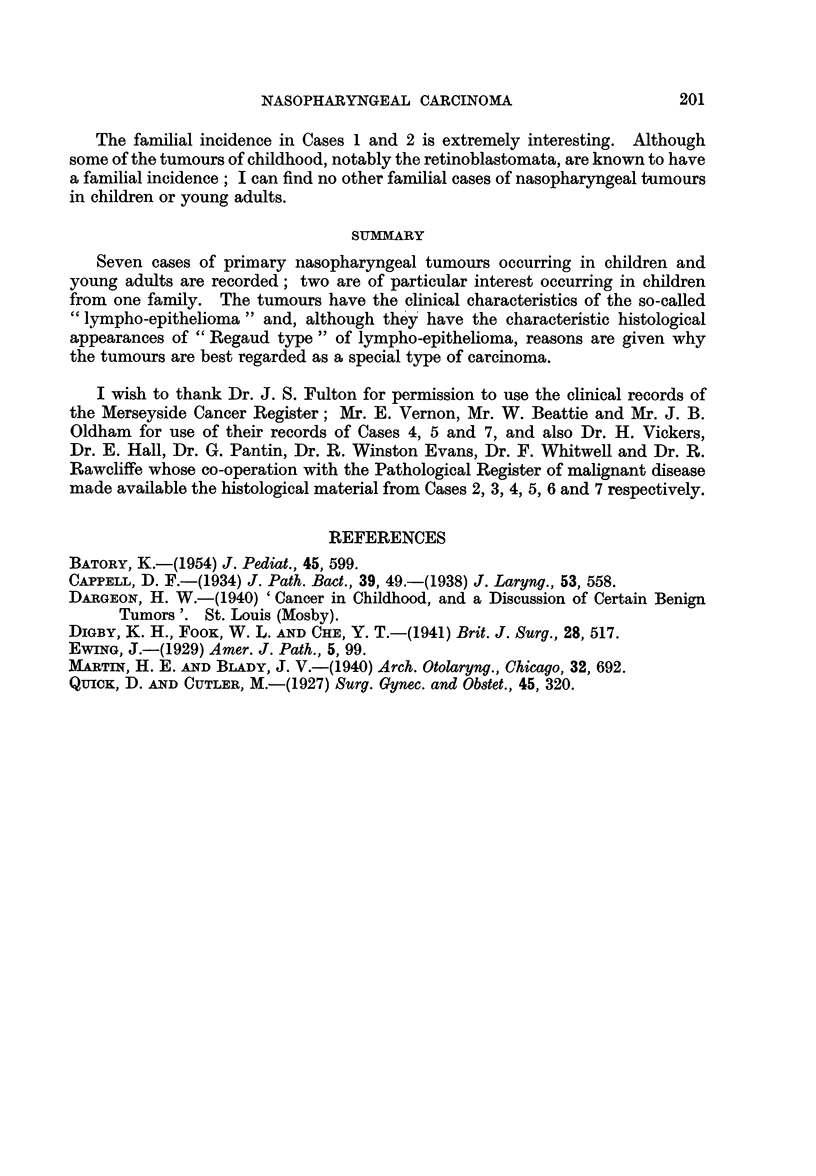# Nasopharyngeal Carcinoma in Children and Young Adults

**DOI:** 10.1038/bjc.1958.22

**Published:** 1958-06

**Authors:** E. M McConnell

## Abstract

**Images:**


### NASOPHARYNGEAL CARCINOMA IN CHILDREN AND YOUNG ADULTS

E. M. McCONNELL

*From the Department of Pathology, Liverpool Radium Institute*

Received for publication March 5, 1958

THE commonest nasopharyngeal tumours occurring in children and young adults are the nasopharyngeal fibroma, the lymphosarcoma and the anaplastic carcinoma or " lympho-epithelioma ". The nasopharygeal fibromas usually run a benign clinical course, they form bulky tumours and give rise to post-nasal obstruction and epistaxis. The lymphosarcomas are rapidly growing bulky malignant tumours which give rise to post-nasal obstruction and pressure symptoms ; however, epistaxis is uncommon ; although extremely radiosensitive the prognosis of nasopharyngeal lymphosarcomas is very poor, metastasis to cervical lymph nodes and lungs occurring early. The anaplastic carcinomas or " lympho-epitheliomas " are characterised by a tendency to spread early to adjacent lymph nodes whilst the primary is still obscure. Like the lymphosarcomas although they are extremely radiosensitive their prognosis is very poor, metastasis occurring to the vertebral column and liver.

It is proposed in this paper to describe the clinical and histological features of 7 cases of nasopharyngeal carcinoma occurring in children and young adults. Two of the cases are of particular interest occurring in siblings.

### CASE REPORTS

### *Case* 1

A female child, 8 years of age, was referred to hospital with general debility, undernourishment, swelling of the cervical glands and of the right side of the face. On examination the child was very undersized for her age. The upper cervical lymph nodes on both sides were enlarged and hard. There was marked swelling of the right cheek, bulging forwards of the soft palate and swelling and ulceration in the posterior part of the upper alveolar sulcus on the right side. There was radiological evidence of malignant invasion of the maxilla and coronoid process of the mandible.

Histological examination of a lymph node excised from the submaxillary area showed the nodal structure to be almost completely obliterated by fibrosis and pyogenic infection. Islets of bizarre malignant cells with large vesicular nuclei, one or more prominent nucleoli, and poorly defined cytoplasmic borders were present in the sinuses (Fig. 1). The cells were considered to be probably carcinomatous in type.

A radical course of radiotherapy resulted in the disappearance of the right sided facial swelling and of the enlarged cervical glands.

The child remained well for 8 months when she developed jaundice as a result of malignant infiltration of the liver. She died 3 months after the onset of jaundice.

Autopsy showed an extremely emaciated and deeply jaundiced child, markedly undersized for her age with a protuberant abdomen and pitting oedema of the ankles. The only evidence of malignancy was in the liver which was grossly enlarged as a result of massive tumour infiltration. The metastatic tumours varied in size from 5 mm. to 10 cm.; they presented an umbilicated appearance at the surface of the liver and were firm to cut. In the treated area there was a thickening of the tissues on the inner aspect of the right mandible and some fibrosis of the neck.

Histologically the tumours in the liver were divided by trabeculae of degenerate liver cells into solid alveoli (Fig. 2). The tumour cells which were smaller than in the biopsy specimen, tended to assume a spindle form and were more definitely carcinomatous in type. Lymphocytic infiltration was not present in the tumours in the liver.

*Case* 2

The younger brother of Case 1 was admitted to hospital at the age of 8 years with a history of (1) general debility and bilateral cervical adenopathy of 3 years' duration, considered at one time to be possibly tuberculous in origin; and (2) proptosis of short duration. On examination the child was markedly undersized for his age, he had bilateral cervical glandular enlargment, bilateral proptosis and a large tumour occupying the nasopharynx and infiltrating the retro-orbital tissues.

Histological examination of biopsy specimens from the nasopharyngeal mass and cervical lymph nodes showed infiltration by an anaplastic tumour composed of sheets and strands of syncytial cells having large oval or spherical nuclei, with one or more prominent nucleoli (Fig. 3). The nuclei were poor in chromatin and in some cases appeared to have undergone hydropic degeneration consisting of a nuclear membrane enclosing the nucleoli. In the sections from the nasopharyngeal tumour the sheets of tumour cells were growing in a fibrous stroma which was infiltrated by lymphocytes, plasma cells and eosinophil leucocytes (Fig. 4). In the lymph nodes the tumour cells were infiltrating the sinuses. In some places they completely replaced the nodal structure, in others they formed anastomosing columns of tumour separated by lymphoid tissue which was infiltrated by plasma cells. Although it was sometimes difficult to decide whether the tumour cells were of epithelial or reticulum type, in parts of the tumour its epithelial nature was obvious.

A radical course of radiotherapy resulted in regression in size of the cervical glands and nasopharyngeal tumour and a marked improvement of the proptosis. However he soon developed jaundice and a palpable liver and died 8 months after treatment.

*Case* 3

A female child 13 years of age was admitted to hospital with a 12 month's history of intermittent deafness and nasal discharge on the right side. Examination showed a nasopharyngeal tumour obstructing the eustachian tube.

Histological examination of a biopsy specimen showed an anaplastic carcinoma with very little stromal reaction but infiltrated with lymphocytes, plasma cells and eosinophils. The tumour cells had a similar appearance to Cases 1 and 2, having a large spherical nucleus with a sharply defined nuclear margin, poor in chromatin and with one or two prominent nucleoli. In some parts the cytoplasmic borders of the cells were distinct, in others a syncytial arrangement was present.

The child was treated by a radical course of radiotherapy with a temporary regression of symptoms. However she died 11 months after treatment with a recurrent nasopharyngeal tumour and metastatic deposits in the liver, vertebral column and skull.

*Case* 4

A female child 13 years of age was admitted to hospital with a history of (1) deafness and nasal discharge on the right side of 12 months' duration and (2) difficulty in swallowing and a sore throat of 3 months' duration.

On examination the child was undersized and had obviously lost weight. The soft palate was bulged forwards by a solid tumour lying behind it and filling the post-nasal space on the right side. At least one large soft fleshy gland was present under the upper end of the sternomastoid, filling the carotid triangle and possibly concealing other glands.

Histological examination of a biopsy specimen from the nasopharyngeal tumour showed an anaplastic tumour consisting of sheets of syncytial cells with large, spherical, bloated hydropic nuclei with one or two nucleoli (Fig. 5 and 6). The sheets of tumour cells were separated by strands of fibrous tissue infiltrated by lymphocytes, plasma cells and occasional eosinophil leucocytes. Although at the surface of the tumour it was possible to trace the continuity of the tumour cells with the epithelium in the deeper parts of the tumour the degree of anaplasia and the extent of hydropic degeneration of the nuclei was such that it was impossible to have told their tissue of origin.

A radical course of radiotherapy resulted in the disappearance of the nasopharyngeal tumour and the enlarged cervical gland with relief of symptoms.

At the time of writing, 12 months after treatment, there is no evidence of any local recurrence. There is, however, a swelling of doubtful nature overlying the sacrum, which shows radiological evidence of invasion of the underlying bone and is presumably a metastatic deposit.

*Case* 5

A young adult male, 17 years of age was referred to hospital with a history of painless, bilateral cervical adenopathy of 1 year duration.

On examination the cervical glands on both sides were enlarged and hard, those on the right side being fixed to the adjacent tissues ; and there was a tumour bulging the posterior pharyngeal wall forwards.

A biopsy excision of one of the left cervical nodes was performed. Histological examination showed that although the nodal architecture was still recognisable there was an extensive infiltration, chiefly in the sinusoids of anaplastic carcinoma (Fig. 7 and 8). The tumour was essentially similar to the previous cases described, although its epithelial nature was more obvious than in some of them ; the syncytial arrangement and hydropic degeneration of nuclei being less marked.

A radical course of radiotherapy resulted in regression of the nasopharyngeal tumour and cervical glands. The patient died 2½ years after treatment, the nasopharyngeal tumour being recurrent and metastatic deposits present in the vertebrae, pelvis, ribs and skull.

*Case* 6

A young adult female, 18 years of age, was referred to hospital with an 8 months' history of trismus. On examination she had bilateral enlarged cervical glands and a tumour in the nasopharynx.

Histological examination of one of the cervical glands showed a similar appearance to the glands examined in Cases 2, 4 and 5. The sinusoids were stuffed with sheets of syncytial cells with large oval vesicular nuclei having one or two nucleoli. A few of the nuclei showed hydropic degeneration. The residual lymphoid tissue which was infiltrated with plasma cells and eosinophil leucocytes formed narrow trabeculae between the anastomosing cords of tumour cells (Fig. 9 and 10).

The patient was treated by a radical course of radiotherapy which resulted in regression of the nasopharyngeal tumour and cervical glands and relief from the trismus. She died 3 years after her first treatment having remained well for 2 years before the nasopharyngeal growth was found to be recurrent. At the time of her death the tumour had spread up through the base of the skull to involve the brain.

*Case* 7

A female, 26 years of age, was referred to hospital with a 14 months' history of intermittent swelling of the left cervical glands. On examination a large mass of mobile glands was found below the angle of the jaw on the left side. In spite of careful examination no primary tumour could be found.

Local excision of the glands was carried out and histological examination showed an appearance almost identical with Case 5. There was an extensive infiltration of the sinusoids by sheets of anaplastic carcinoma cells some of which assumed a syncytial form, but mostly their cytoplasmic borders were distinct. The sheets of tumour cells were separated by trabeculae of compressed lymphoid tissue infiltrated by plasma cells and occasional eosinophil leucocytes.

Eight months after the local excision the patient received a radical course of radiotherapy to recurrent glands. Following radiotherapy she remained well for almost a year, when recurrent cervical glands necessitated block excision of the glands first of the left and then of the right side, palliative radiotherapy was given to metastatic deposits in the liver.

The patient died 3 years and 9 months after the original local excision, with recurrent tumour in the neck and metastatic deposits in the liver.

### DISCUSSION

Primary malignant nasopharyngeal tumours are uncommon except in some parts of the far East, where they " may rank second in frequency in a list of all malignant diseases " (Martin and Blady, 1940; Digby, Fook and Che, 1941). In the area covered by the Merseyside Cancer Registration scheme primary nasopharyngeal tumours form 0·2 per cent of the total number of cases of malignant disease registered. There appears, however, to be a relatively high incidence of nasopharyngeal tumours in children and young adults in whom they form a fairly large percentage of the total number of cases of malignant disease. Ewing (1929) stated that carcinoma of the nasopharynx frequently occurs in young subjects between the ages of 10 and 20 years. Martin and Blady (1940) stated that " cancer of the nasopharynx occurs at an earlier age and is found more often in children and at ages below 30 than any other malignant growth of the upper respiratory and alimentary tracts ", 9 per cent of their cases of nasopharyngeal tumours occurred under the age of 15 years, and 18 per cent under the age of 30 years. Dargeon (1940) in a series of 218 cases of cancer in children observed 7 primary nasopharyngeal tumours ; and Digby, Fook and Che (1941), in an analysis of 114 cases of nasopharyngeal carcinoma, also found that the disease occurs not infrequently in children and young adults.

The commonest type of malignant nasopharyngeal tumour which is reported in children and young adults appears to be the so-called " Regaud " or epithelial type of lympho-epithelioma. Cappell (1934, 1938) in his papers on nasopharyngeal tumours included 9 cases of lympho-epithelioma of the nasopharynx, 3 of whom were under the age of 25 years. He placed these 3 cases into the so-called " Regaud type of lympho-epithelioma " group which he considered to be frankly carcinomatous. The tumours were characterised by strands or sheets of epithelial cells, often syncytial in arrangement with large pale staining vesicular or sometimes bloated nuclei, embedded in a fibrous stroma more or less rich in lymphocytes. Ewing (1929) and Dargeon (1940) described their cases of nasopharyngeal cancer in children as lympho-epitheliomas or transitional cell carcinomas. Batory (1954) reported a nasopharyngeal carcinoma in a female child 10 years of age. Biopsy examination of the primary tumour and of the metastatic cervical nodes showed a lympho-epithelioma having a thin collagenous stroma infiltrated by lymphocytes and islands of large reticulum cells.

The histological features and clinical characteristics of the 7 cases reported in this paper are similar to those described by Ewing, Cappell, Dargeon and Batory. From the pathological point of view these tumours would appear to have an epithelial origin. Their mode of spread first to local lymph nodes and then to the liver and skeletal system is that of a carcinoma, as is their mode of growth within the lymph nodes where they infiltrate the sinuses. The " intimate " intermingling with lymphocytes is probably fortuitous, the nasopharynx in children and young adults being characterised by abundant lymphoid tissue. Within the lymph nodes the lymphocytes are mostly present as compressed cords of residual lymphoid tissue between the sheets of anaplastic tumour cells rather than diffusely intermingled with them. In the present series it was possible in one case to examine metastatic deposits outside the lymphoid system, i.e. the liver. The deposits in the liver consisted of one cell type only, the anaplastic epithelial cell, and lymphocytes were absent.

Although probably epithelial in origin these tumours are not typical squamous carcinomas either clinically or histologically. They occur at an earlier age, are more radiosensitive and metastasise earlier than squamous lesions, and histologically they never show any evidence of intercellular bridges, keratinisation or epithelial pearl formation. They have certain similarities to the transitional cell epidermoid carcinomas described by Quick and Cutler (1927) ; both are radiosensitive and histologically in both types " spines, hornification and pearl formation are regularly absent ". However Quick and Cutler describe their tumours as being composed of " small cells uniform in size with a relatively large hyperchromatic nucleus and scanty cytoplasm. They are closely packed, with little intercellular substance. The cells sometimes form solid groups, sometimes they grow in anastomosing columns of opaque granular cells with convolutions." This description does not quite fit with the tumours described in this paper.

The mucosa of the nasopharynx is derived from the entoderm of the primitive gut. It is of several types—pseudostratified ciliated columnar, stratified columnar and stratified squamous with both mucous and mixed glands. It is possible that the nasopharyngeal tumours in children and young adults arise from the columnar epithelium. Cells with very similar features to the better differentiated tumour cells can be seen in the pseudostratified columnar epithelium lining the ducts from the submucosal glands. The fact that with increasing age the ciliated pseudostratified columnar epithelium is replaced by stratified squamous over large areas may explain the preponderance of these tumours in the younger age group.

Clinically the primary tumours are characteristically symptomless in the early stages and the commonest initial symptom is painless swelling of the cervical glands. In a few cases the initial symptoms are due to the presence of the nasopharyngeal tumour—obstruction to the eustachian tube giving rise to deafness, involvement of the adjacent nerves giving a neuralgic type of pain, and involvement of the pterygoid muscles causing trismus. In the present series painless cervical adenopathy was the initial symptom in 5 cases, in one of which it was associated with trismus. In the other 2 cases intermittent deafness and nasal obstruction were the initial symptoms. In 6 of the cases careful examination revealed the presence of the nasopharyngeal tumour.

In the treatment of the tumours radiotherapy holds out the best hope of cure. In view of the inaccessible site of the primary growth and the extensive metastatic deposits which are present in the cervical glands before the diagnosis is made surgical treatment is usually limited to diagnostic biopsy procedures.

Although extremely radiosensitive the prognosis of nasopharyngeal carcinomas in children and young adults is very bad, the average survival being about 2 years from the onset of symptoms. In the present series one patient is still alive 12 months after treatment but with a swelling over her sacrum involving the bone. The average survival time in the other 6 cases was 2 years after treatment or 3 years after the onset of symptoms. Local recurrence occurred in 5 cases in 2 of which extension to the brain was present. Metastatic deposits were present in the liver in 4 cases and in the skeletal system in 2. The prognosis would probably be improved if the diagnosis was made earlier in the course of the disease, the average time interval between the initial symptoms and diagnosis being 14 months.

---

### EXPLANATION OF PLATES

Fig. 1.—Islet of tumour cells within the sinus of a submaxillary lymph node. Case 1. × 260.
Fig. 2.—Metastatic deposit in the liver. Case 1. × 65.
Fig. 3.—Nasopharyngeal tumour showing typical syncytial mass of cells with characteristic nuclei. Case 2. × 260.
Fig. 4.—Nasopharyngeal tumour. Case 2. × 65.
Fig. 5.—Nasopharyngeal tumour. Case 4. × 65.
Fig. 6.—As Fig. 5 showing an area of extensive hydropic nuclear degeneration giving rise to a " bloated " appearance of the nuclei. ×260.
Fig. 7.—Cervical lymph node showing infiltration of sinuses by tumour. Case 5. × 65.
Fig. 8.—As Fig. 7. × 260.
Fig. 9.— Cervical lymph node showing infiltration of sinuses by tumour. Case 6. × 65.
Fig. 10 —As Fig 9. × 260.

1

2

3

4

5

McConnell.

6

7

8

9

10

The familial incidence in Cases 1 and 2 is extremely interesting. Although some of the tumours of childhood, notably the retinoblastomata, are known to have a familial incidence ; I can find no other familial cases of nasopharyngeal tumours in children or young adults.

### SUMMARY

Seven cases of primary nasopharyngeal tumours occurring in children and young adults are recorded ; two are of particular interest occurring in children from one family. The tumours have the clinical characteristics of the so-called " lympho-epithelioma " and, although they have the characteristic histological appearances of " Regaud type " of lympho-epithelioma, reasons are given why the tumours are best regarded as a special type of carcinoma.

I wish to thank Dr. J. S. Fulton for permission to use the clinical records of the Merseyside Cancer Register ; Mr. E. Vernon, Mr. W. Beattie and Mr. J. B. Oldham for use of their records of Cases 4, 5 and 7, and also Dr. H. Vickers, Dr. E. Hall, Dr. G. Pantin, Dr. R. Winston Evans, Dr. F. Whitwell and Dr. R. Rawcliffe whose co-operation with the Pathological Register of malignant disease made available the histological material from Cases 2, 3, 4, 5, 6 and 7 respectively.

### REFERENCES

Batory, K.—(1954) *J. Pediat.*, **45**, 599.
Cappell, D. F.—(1934) *J. Path. Bact.*, **39**, 49.—(1938) *J. Laryng.*, **53**, 558.
Dargeon, H. W.—(1940) 'Cancer in Childhood, and a Discussion of Certain Benign Tumors'. St. Louis (Mosby).
Digby, K. H., Fook, W. L. and Che, Y. T.—(1941) *Brit. J. Surg.*, **28**, 517.
Ewing, J.—(1929) *Amer. J. Path.*, **5**, 99.
Martin, H. E. and Blady, J. V.—(1940) *Arch. Otolaryng., Chicago*, **32**, 692.
Quick, D. and Cutler, M.—(1927) *Surg. Gynec. and Obstet.*, **45**, 320.